# A single-site retrospective cohort on the profile of in-hospital stroke: an alarming sentinel of systemic failures

**DOI:** 10.1055/s-0046-1820524

**Published:** 2026-05-12

**Authors:** Ana Beatriz Marangoni Baston, Pedro Machry Pozzobon, Italo Merino Loes, Gabriel Pinheiro Modolo, Daniel Fabiano Barbosa dos Santos, Natália Cristina Ferreira, Silméia Garcia Zanati Bazan, Gustavo José Luvizutto, Rodrigo Bazan, Cristiane Mendes-Chiloff

**Affiliations:** 1 Universidade Estadual Paulista, Faculdade de Medicina de Botucatu, Unidade de Acidente Vascular Cerebral, Botucatu SP, Brazil.; 2 Universidade Estadual Paulista, Faculdade de Medicina de Botucatu, Departamento de Clínica Médica, Botucatu SP, Brazil.; 3 Universidade Federal do Triângulo Mineiro, Departamento de Fisioterapia Aplicada, Uberaba MG, Brazil.; 4 Universidade Estadual Paulista, Faculdade de Medicina de Botucatu, Departamento de Neurociências e Saúde Mental, Botucatu SP, Brazil.

**Keywords:** Developing Countries, Time-to-Treatment, Health Services Accessibility, Early Diagnosis

## Abstract

**Background:**

In-hospital stroke is associated with delayed recognition, reduced access to acute treatment, and worse outcomes, particularly in low- and middle-income countries.

**Objective:**

To characterize the clinical profile, care processes, and outcomes of patients with ischemic stroke occurring during hospitalization.

**Methods:**

The present retrospective cohort study included adult patients with ischemic stroke diagnosed during hospitalization at a comprehensive stroke center. Stroke recognition time was used as the reference point. Clinical characteristics, stroke severity, acute treatment, and in-hospital outcomes were analyzed. Primary outcomes were in-hospital mortality, discharge destination, and functional independence.

**Results:**

Fifty-two patients were included. Most strokes occurred in patients admitted for non-neurological conditions, mainly in surgical specialties. Stroke recognition was delayed in a relevant proportion of cases, and reperfusion therapy was infrequently performed, with no procedure-related complications. Moderate-to-severe strokes were common. In-hospital mortality was 9.6%, and functional status significantly declined from admission to discharge. Most patients received therapeutic care planning and were referred for rehabilitation.

**Conclusion:**

In-hospital ischemic stroke predominantly affects patients hospitalized for non-neurological conditions and is frequently recognized late, limiting access to reperfusion therapies. These findings highlight important gaps in in-hospital stroke detection and support the implementation of structured rapid-response protocols.

## INTRODUCTION

In-hospital stroke is defined as an acute cerebrovascular event that occurs during hospitalization of a patient who was initially admitted for a different medical condition. These account for a substantial proportion of all stroke cases in the population. 1 2 Studies indicate that between 6.5 and 15% of all stroke patients experience symptom onset while already hospitalized, and in-hospital stroke complicates approximately 0.04 to 0.06% of all hospital admissions. 1 3 4

In-hospital stroke is generally more severe than community-onset stroke and is associated with poor quality of stroke care, increased hospitalization costs, and higher morbidity and mortality rates. 2 3 The demographic characteristics of patients with in-hospital stroke were similar to those of patients with community-onset stroke; however, a higher prevalence of comorbidities and lower baseline functional status were important predictors of poor prognosis. 5

Approximately half of the in-hospital stroke events occur in patients admitted for surgical procedures or invasive diagnostic examinations. 4 Timely recognition of stroke symptoms is crucial, as stroke is a time-dependent condition in which early treatment significantly affects outcomes. 6 Paradoxically, patients with an in-hospital stroke often experience delays in the recognition of initial symptoms and evaluation by a neurologist. 3 7 Moreover, these patients frequently present contraindications to systemic thrombolysis with recombinant tissue plasminogen activator, making mechanical thrombectomy the only safe reperfusion therapy in many cases. 1 5 8 9 10 11

Evidence suggests that delays in symptom recognition and specialist evaluation reflect failures in healthcare delivery. 2 The current study aimed to characterize the profile of patients diagnosed with in-hospital stroke in terms of their pre-stroke functional status and comorbidities. Additionally, this study sought to assess the quality-of-care indicators related to stroke management in a hospital setting, identify the types of hospitalization most associated with stroke occurrence, and evaluate the clinical outcomes of these patients.

## METHODS

### Study design, setting, and participants

The present retrospective cohort study used a stroke database from the electronic medical records of patients hospitalized at the Stroke Unit of the Teaching Hospital of Faculdade de Medicina de Botucatu, Brazil, between July 2019 and December 2024. Patients of both sexes who experienced a stroke in a hospital setting during the study period and whose data were correctly logged into the database were included. The study was approved by the Institutional Ethics Committee (approval number: 6.334.539).

The study included patients aged > 18 years with in-hospital ischemic or hemorrhagic stroke confirmed by neuroimaging. Individuals were excluded if they presented with other neurological diseases as confirmed by computed tomography or magnetic resonance imaging. All patients were admitted within the first 48 hours after ictus or were referred to the hospital due to clinical severity or instability requiring intensive care support. The patients were followed up at the cerebrovascular disease outpatient clinic 90 days after admission.

### Data collection

Information was retrieved from the stroke databank, created by completing the hospital discharge form from the electronic medical records of patients with in-hospital stroke during the study period.

### Patient characteristics

The patient characteristics included age, sex, race, and body mass index. Stroke severity was measured using the National Institutes of Health Stroke Scale (NIHSS) 12 at the onset of stroke. The NIHSS scores were stratified as mild (NIHSS 1–7), moderate (NIHSS 8–16), or severe (NIHSS >16). 13 Comorbid illness included atrial fibrillation/flutter, prosthetic heart valve, previous stroke/transient ischemic attack, coronary artery disease/previous myocardial infarction, carotid stenosis, diabetes mellitus, peripheral vascular disease, hypertension, smoking, dyslipidemia, and heart failure. The classes of medications used before admission included antiplatelet, anticoagulant, antihypertensive, cholesterol-reducing, and diabetic medications. Prior modified Rankin Scale (mRS) 12 status was documented before admission. The mRS is an ordinal scale ranging from 0 (no disability) to 5 (severe disability), with a score of 6 assigned to those who died.

### Clinical information

Clinical information recorded included intravenous tissue plasminogen activator (tPA) treatment, early antithrombotic, deep venous thrombosis prophylaxis for non-ambulatory patients, antithrombotic therapy on discharge, anticoagulation on discharge for atrial fibrillation/flutter, statin therapy, and smoking cessation counseling if appropriate on discharge. Quality measures included cholesterol treatment if low-density lipoprotein level was > 100 mg/dL or not documented; dysphagia screening; stroke education; and rehabilitation assessment. For in-hospital stroke, the documented time of symptom recognition was used as the onset, and the time of stroke-team activation or initial neurological evaluation was considered the door. These onset-to-door analyses were applied only to patients with confirmed ischemic stroke. Examinations performed during hospitalization were also documented, including the type of stroke, Bamford topography, 14 and etiology (based on the Trial of Org 10172 in Acute Stroke Treatment [TOAST] classification). 15

### Outcome measures

The primary outcome measures were in-hospital mortality, discharge to home, and independent ambulation at discharge. Symptomatic intracranial hemorrhage was an additional primary outcome of interest in a subset of patients who received intravenous tPA. Additional complications of tPA that were recorded included serious bleeding within 36 h of treatment or other serious complications that required additional medical interventions or a prolonged length of stay.

### Statistical analysis

All data were analyzed using the IBM SPSS Statistics for Windows (IBM Corp.), version 20.0. For the descriptive analysis, means, medians, standard deviations, and 95% confidence intervals were calculated. The prevalence and frequencies were expressed as percentages, and appropriate tables were designed to describe the study population.

## RESULTS

Among the 68 patients initially screened for suspected in-hospital stroke, 10 were classified as stroke mimics and excluded. Additionally, 5 intracerebral hemorrhage and 1 subarachnoid hemorrhage were identified and excluded, resulting in a final cohort of 52 patients with confirmed ischemic stroke. The majority were male (55.7%), over 60 years of age (67.3%), and had either a low (32,.7%) or intermediate (40.4%) level of schooling. Additionally, 57.7% reported having a partner, and 57.7% held low-skilled occupations ( **Supplementary Material Table S1** –available at https://www.arquivosdeneuropsiquiatria.org/wp-content/uploads/2026/04/ANP-2025.0317-Supplementary-Material.docx ).

The most prevalent comorbidities were systemic arterial hypertension (71.2%), diabetes mellitus (40.4%), and dyslipidemia (32.7%). Moreover, 38,5% of the patients had 3 or more preexisting conditions. The regular use of antihypertensive agents (63.5%), statins (50.0%), and antiplatelet medications (75.0%) was also observed. Most admissions occurred in surgical specialties (45.6%), with 17.6% of patients admitted through the emergency department for diagnostic investigations ( Table 1 ).

**Table 1:** Hospitalization characteristics and stroke-specific clinical classifications

	Variables		N	%
**Hospitalization characteristics**	Assessment pathway	Stroke Unit	05	9.6
		Ward	09	17.3
		Neurology consultation	26	50.0
		Emergency	03	5.7
		Informal identification	04	7.7
	Type of admission	Surgical	19	36.5
		Clinical	21	40.4
		Emergency	12	23.1
	Onset-to-door time*	< 4 h	31	59.6
		4–12 h	08	15.4
		13–24h	06	11.5
		25–36h	02	3.8
		≥ 36h	05	9.6
	Treatment	Conservative	40	76.9
		Intravenous thrombolysis	03	3.8
		Thrombectomy	07	13.5
		Not applicable	02	3.8
	Discharge destination	Home	42	80.8
		Ward	04	7.7
		ICU	01	1.9
		Death	05	9.6
**Stroke characteristics**	TOAST	Large atherosclerosis	06	11.5
		Cardioembolism	05	9.6
		Small vessel occlusion	03	5.8
		Other determined etiology	18	34.6
		Undetermined etiology	15	28.8
		Not applicable	03	9.6
	Bamford	LACS	15	28.8
		TACS	14	26.9
		PACS	12	23.1
		POCS	08	15.4
		Not applicable	03	5.8
	Number of symptoms	None	03	5.8
		1–2	19	36.5
		3–4	23	44.2
		5–6	07	13.5

Abbreviations: ICU, intensive care unit; LACS, lacunar syndrome; PACS, partial anterior circulation; POCS, posterior circulation; TACS, total anterior circulation; TOAST, Trial of Org 10172 in Acute Stroke Treatment.

Note: *‘onset’ was defined as the documented time when neurological symptoms were first recognized by clinical staff, and ‘door’ was defined as the time of stroke team activation or initial neurological evaluation.

Among patients with suspected stroke, 45.6% were referred for neurological consultation. The most frequently reported symptoms were hemiparesis (67.3%), aphasia (46.1%), and dysarthria (44.2%). Furthermore, 57.7% of patients exhibited 3 or more neurological signs. Notably, 59.6% of patients were assessed within the so-called “therapeutic window,” that is, within the first 4 h after symptom onset, allowing for thrombolysis when indicated. Additionally, 86.5% of the patients were evaluated within 24 h of symptom onset, which is the maximum time window for mechanical thrombectomy. However, in 13.4% of suspected cases, stroke was not identified within 24 h, resulting in missed opportunities for reperfusion therapy ( Table 1 ).

Most patients (76.9%) received conservative treatment; only 17.6% underwent reperfusion therapy (either thrombolysis or mechanical thrombectomy). No procedure-related complications were identified among patients who received cerebral reperfusion therapy. According to the Bamford classification, most patients (65.4%) presented with clinical features suggestive of anterior circulation involvement. Based on etiological investigation, the most common TOAST classification observed was “´other determined etiology” (34.6%), typically related to medical procedures ( Table 1 ).

**Figure 1:**
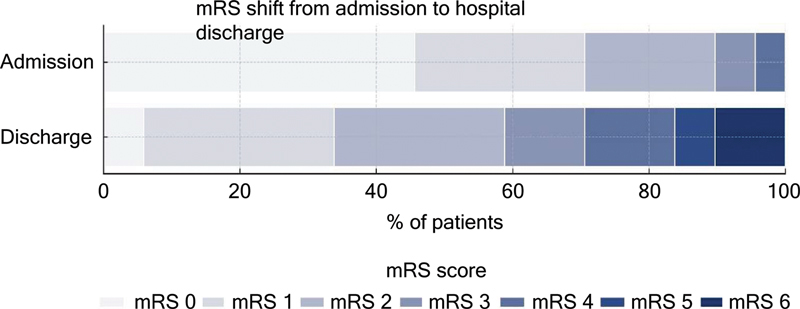
Distribution of modified Rankin Scale (mRS) scores at admission and hospital discharge.

Regarding the clinical scales used to assess patients with suspected stroke ( Table 2 ), 44.2% presented with a baseline NIHSS score classified as moderate-to-severe. In terms of functional status, 75% of the patients were previously independent (mRS 0–2). The overall mortality rate was 9.6%. At discharge, only 28.8% of the patients remained functionally independent, with the remainder exhibiting functional impairments. A significant decline in functional status was observed when the initial and discharge mRS scores were compared ( *p* < 0.0001) ( Figure 1 ). At the time of discharge, 92.3% of patients underwent therapeutic care planning, and 90.4% were referred for rehabilitation.

**Table 2:** Clinical outcomes comparing admission and discharge

Scale/category		Admission, n (%)	Discharge, n (%)
NIHSS	Mild (≤ 7)	29 (55.9)	38 (73.1)
	Moderate (8–15)	12 (23.1)	06 (11.5)
	Severe (≥ 16)	11 (21.1)	08 (15.4)
mRS	0	24 (46.1)	01 (2.3)
	1	15 (28.9)	14 (26.9)
	2	07 (13.5)	14 (26.9)
	3	03 (5.7)	07 (13.5)
	4	03 (5.7)	08 (15.4)
	5	−	03 (5.8)
	6 (death)*	−	05 (9.6)

Abbreviations: mRS, modified Rankin Scale; NIHSS, National Institutes of Health Stroke Scale.

Note: Stroke score: *Stroke-associated pneumonia (n = 3), refractory septic shock of pulmonary origin (n = 1), septic shock of cutaneous origin (n = 1), and upper gastrointestinal bleeding (n = 1).

## DISCUSSION

The present retrospective cohort study showed that in-hospital stroke predominantly affects independent function and autonomy in older adults admitted for non-neurological conditions, primarily for surgical procedures. Additionally, a significant proportion of patients experience delays in stroke recognition and intervention. Low rates of cerebral reperfusion therapy were also observed in this sample, highlighting gaps in in-hospital stroke detection and fast-response teams.

An unexpected profile was observed among patients with in-hospital stroke, with most cases occurring in individuals without prior disability or with minimal functional impairment (mRS 0–1). Additionally, most patients were hospitalized for non-neurological reasons, mainly in surgical wards. This patient group is typically not prioritized for neurological monitoring under standard hospital protocols. In surgical settings, patient monitoring is generally focused on immediate postoperative parameters, such as hemodynamics, bleeding, and infection, 16 with no routine assessment for new neurological signs. Cumbler (2015) 2 and Schürmann et al. (2016) 5 reported that failures or delays in recognizing in-hospital strokes are not due to clinical complexity, but rather the absence of structured neurological vigilance.

Another important outcome was the delay in patients receiving specific cerebral reperfusion interventions. Several aspects need to be considered when highlighting this aspect, such as systemic failure in intrahospital communication and early detection of cases. Nouh et al. (2022) 17 highlighted that although these patients are in a monitored environment, their assessment and treatment are often delayed compared to patients presenting to the emergency department, contributing to higher morbidity and mortality rates. Furthermore, we highlight the need for greater interprofessional integration with structured neurological surveillance protocols regardless of the cause of hospitalization. However, it is worth noting that a large proportion of our sample comprised stroke mimics, which may have made recognition difficult. Suspected stroke symptoms in hospitalized patients are often non-focal and can be confounded by medication, metabolic encephalopathy, and comorbid illness. 17

In high-income countries (HICs), in-hospital stroke accounts for approximately 5 to 17% of all acute stroke admissions and generally occurs in neurology units. In this context, the presence of organized in-hospital stroke code protocols has been associated with higher reperfusion rates and better functional outcomes. 7 17 18 In contrast, our single-center cohort from a low- and middle-income country (LMIC) showed a different pattern. Most strokes occurred in non-neurology wards, where neither structured neurological surveillance nor rapid-response pathways were in place, resulting in organizational delays and low cerebral reperfusion rates, even among individuals within the appropriate therapeutic window. These findings suggest that the observed differences are largely systemic and structural in nature, highlighting the need for low-cost neurological screening tools adapted to resource-limited environments.

Finally, a significant proportion of patients progressed to severe disability (mRS 4–5) or death (mRS 6), underscoring the negative impact of in-hospital stroke on short-term functional outcomes. Compared with community-onset stroke, in-hospital stroke represents a distinct stroke subgroup with poorer outcomes at hospital discharge 19 20 and increased in-hospital mobility after adjusting for age, sex, and cardiovascular risk factors. 20 Delays in initiating stroke interventions may have an impact on long-term functionality. Strategies such as cerebral reperfusion therapies, use of anticoagulants/antiplatelet agents, 21 and early rehabilitation 22 are widely recognized for reducing disability and promoting the autonomy of individuals after stroke.

One of the strengths of the current study is the comprehensive characterization of in-hospital stroke in a low- and middle-income setting, encompassing pre-stroke functional status, comorbidities, admission context, and quality of care metrics. A comprehensive, semi-automated stroke center database provided diagnostic rigor and minimized data loss. However, the single-center study may limit external validity, and the retrospective design may reduce the accuracy of clinical records. The modest sample size reduced the statistical power for subgroup analyses and restricted the identification of determinants of late recognition or adverse outcomes. Furthermore, the lack of longitudinal follow-up did not allow for the assessment of sustained functional decline or post-discharge mortality.

### Implications for policy, practice, and research

In general, the occurrence of in-hospital stroke can be considered a marker of systemic failure and should be proposed as an indicator of hospital care quality. Electronic medical records should incorporate neurological alerts and simple digital neurological screening tools that can be used by general practitioners for systematic neurological assessment. Implementing pragmatic and low-cost strategies in LMICs is essential to reduce delays in the recognition and management of in-hospital stroke. Hospitals should adopt routine bedside neurological checklists administered by non-neurology staff, establish structured in-hospital stroke code protocols to facilitate rapid response, and conduct regular interdisciplinary training sessions focused on the early detection of acute neurological changes.

The development of low-cost support systems or artificial intelligence-based stroke recognition algorithms embedded in electronic health systems may offer viable paths forward. 23 The current study highlights three points:

- in-hospital stroke is a sentinel event that requires a rapid response systems; 17
- sentinel events in acute care expose systemic vulnerabilities that compromise timely neurological evaluation; 3 17
- unrecognized in-hospital stroke should be regarded as a sentinel indicator of failure in care delivery. 3

In conclusion, in-hospital stroke often affects patients who were previously independent and admitted for non-neurological reasons, who represent a group typically excluded from neurological surveillance. Despite timely symptom onset in many cases, delays in recognition and low treatment rates reveal institutional gaps in acute stroke response. These findings underscore the need for structured neurological monitoring across all hospital units and reinforce the role of in-hospital stroke as a sentinel event indicating systemic vulnerability.
